# Identifying Asthma-Related Symptoms From Electronic Health Records Using a Hybrid Natural Language Processing Approach Within a Large Integrated Health Care System: Retrospective Study

**DOI:** 10.2196/69132

**Published:** 2025-05-02

**Authors:** Fagen Xie, Robert S Zeiger, Mary Marycania Saparudin, Sahar Al-Salman, Eric Puttock, William Crawford, Michael Schatz, Stanley Xu, William M Vollmer, Wansu Chen

**Affiliations:** 1 Department of Research and Evaluation, Kaiser Permanente South California, 100 S Los Robles Ave, 2nd Floor, Pasadena, CA, 91101, United States, 1 626-564-3294; 2 Department of Allergy, Kaiser Permanente South California, San Diego, CA, United States; 3 Department of Clinical Science, Kaiser Permanente Bernard J. Tyson School of Medicine, Pasadena, CA, United States; 4 Department of Allergy, Kaiser Permanente South California, Harbor City, CA, United States; 5 Kaiser Permanente Center for Health Research, Portland, OR, United States

**Keywords:** asthma, symptom extraction, electronic health record, natural language processing, transformer-based algorithm, rule-based algorithm

## Abstract

**Background:**

Asthma-related symptoms are significant predictors of asthma exacerbation. Most of these symptoms are documented in clinical notes in a free-text format, and effective methods for capturing asthma-related symptoms from unstructured data are lacking.

**Objective:**

The study aims to develop a natural language processing (NLP) algorithm for identifying symptoms associated with asthma from clinical notes within a large integrated health care system.

**Methods:**

We analyzed unstructured clinical notes within 2 years before a visit with asthma diagnosis in 2013‐2018 and 2021‐2022 to identify 4 common asthma-related symptoms. Related terms and phrases were initially compiled from publicly available resources and then refined through clinician input and chart review. A rule-based NLP algorithm was iteratively developed and refined via multiple rounds of chart review followed by adjudication. Subsequently, transformer-based deep learning algorithms were trained using the same manually annotated datasets. A hybrid NLP algorithm was then generated by combining rule-based and transformer-based algorithms. The hybrid NLP algorithm was finally applied to the implementation notes.

**Results:**

A total of 11,374,552 eligible clinical notes with 128,211,793 sentences were analyzed. After applying the hybrid algorithm to implementation notes, at least 1 asthma-related symptom was identified in 1,663,450 out of 127,763,086 (1.3%) sentences and 858,350 out of 11,364,952 (7.55%) notes, respectively. Cough was the most frequently identified at both the sentence (1,363,713/127,763,086, 1.07%) and note (660,685/11,364,952, 5.81%) levels, while chest tightness was the least frequent at both the sentence (141,733/127,763,086, 0.11%) and note (64,251/11,364,952, 0.57%) levels. The frequency of multiple symptoms ranged from 0.03% (36,057/127,763,086) to 0.38% (484,050/127,763,086) at the sentence level and 0.10% (10,954/11,364,952) to 1.85% (209,805/11,364,952) at the note level. Validation against 1600 manually annotated clinical notes yielded a positive predictive value ranging from 96.53% (wheezing) to 97.42% (chest tightness) at the sentence level and 96.76% (wheezing) to 97.42% (chest tightness) at the note level. Sensitivity ranged from 93.9% (dyspnea) to 95.95% (cough) at the sentence level and 96% (chest tightness) to 99.07% (cough) at the note level. All 4 symptoms had *F*_1_-scores greater than 0.95 at both the sentence and note levels, regardless of NLP algorithms.

**Conclusions:**

The developed NLP algorithms could effectively capture asthma-related symptoms from unstructured clinical notes. These algorithms could be used to facilitate early asthma detection and predict exacerbation risk.

## Introduction

Asthma is a chronic respiratory condition characterized by airway inflammation and obstruction [1], affecting an estimated 262 million people in 2019 worldwide [2]. In the United States, asthma prevalence has increased since the early 1980s, reaching 7.8% in 2020 [3]. Uncontrolled asthma poses a significant health risk to patients and an economic burden to society [4]. Achieving and maintaining asthma control is critical for preventing asthma exacerbation [5].

Asthma diagnosis, control classification, and severity assessment rely on symptom documentation in electronic health records (EHRs), including cough, dyspnea, wheezing, and chest tightness [6]. However, identifying symptoms from EHR is challenging because they are often recorded in free-text clinical notes rather than standardized coding formats.

Natural language processing (NLP) is a computational technique that processes unstructured text data for information extraction, classification, and prediction [7]. NLP has been successfully applied to extract symptoms from clinical narratives using rule-based methods [8-14], and machine learning models [15,16]. Early NLP applications relied on rule-based approaches, whereas recent methods leverage advanced transformer-based deep learning models, such as Bidirectional Encoder Representations from Transformers (BERT) [17], which enhance performance through word embeddings and attention mechanisms [18]. Previous studies have successfully applied NLP to identify asthma diagnosis [19-22], asthma prognosis [23], asthma predictive index [24], asthma control factor [25], and clinician adherence to asthma treatment guidelines [26] among pediatric asthma populations. However, to the best of our knowledge, no previous studies have systematically analyzed asthma symptoms in adult asthma populations using a hybrid NLP approach.

This study aims to develop and validate a hybrid NLP algorithm that combines rule- and transformer-based deep learning approaches to capture 4 common asthma-related symptoms within the EHR of a large integrated health system.

## Methods

### Study Setting

**Figure 1.**
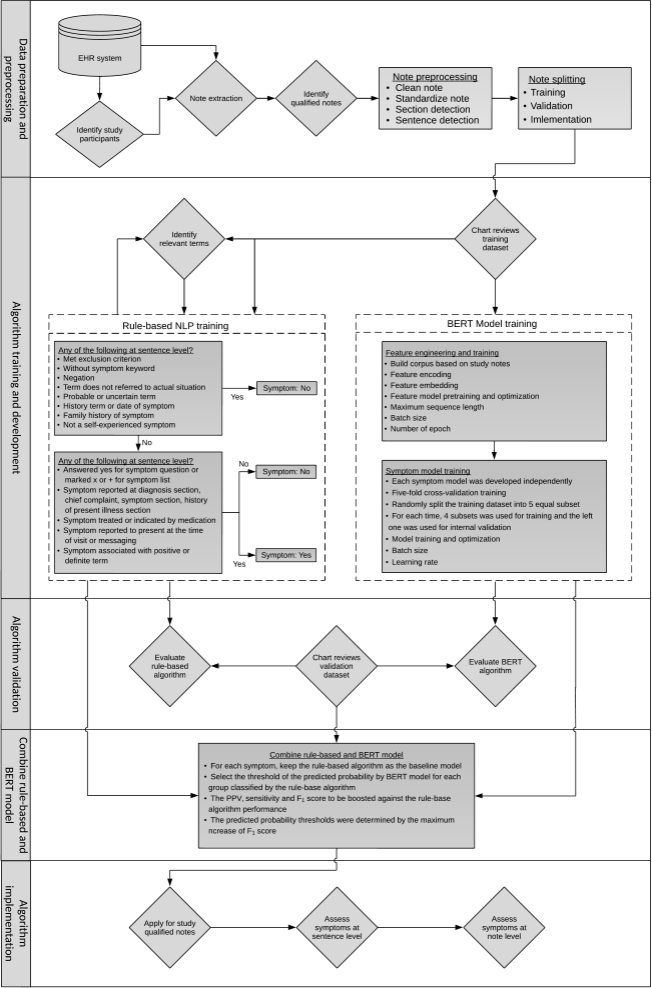
Schematic diagram describing the process for identifying asthma-related symptoms from electronic health records. BERT: Bidirectional Encoder Representations from Transformers; EHR: electronic health record; NLP: natural language processing; PPV: positive predictive value.

This retrospective study was conducted within the Kaiser Permanente Southern California (KPSC), an integrated health care system that provides comprehensive medical services for more than 4.8 million enrollees across 15 large medical centers and over 250 medical offices throughout the Southern California region. The KPSC patient population is demographically representative of Southern California residents [27]. Enrollees obtain their health insurance through group plans, individual plans, Medicare, and Medicaid programs and represent >260 ethnicities and >150 spoken languages. KPSC’s extensive EHR contains structured data (including encounter diagnosis codes and procedure codes, medication dispensing records, immunization records, laboratory results, and pulmonary function test results) and unstructured data (including free-text clinical notes, hospital discharge notes, patient and provider messages, radiology reports, and pathology reports). KPSC’s EHR covers all medical visits across all health care settings (eg, outpatient, inpatient, emergency department, and virtual). The flow of the entire study process is shown in Figure 1, and each step is described in detail below.

### Study Population

The analyses were conducted on a cohort of adult patients who met the study-defined criteria for mild asthma. Eligible patients had a qualifying health care visit with an asthma diagnosis in 2013‐2018 and 2021‐2022. Data from 2019‐2020 were excluded due to health care disruptions during the COVID-19 pandemic [28]. The definition of mild asthma was previously described [29]. Specifically, the participants included patients who (1) were 18-85 years of age with an asthma diagnosis visit (*International Classification of Diseases* [*ICD*]*-9*: 493; *ICD-10*: J45) [index date], (2) had no more than one asthma controller or 2 canisters of short-acting beta2-agonist dispensed in one year prior to or on the index date (aka the baseline window), (3) had no more than 1 acute asthma exacerbation in the baseline window, (4) had no asthma hospitalization or encounter diagnosis of chronic obstructive pulmonary disease, reactive airways dysfunction syndrome, cystic fibrosis, HIV infection, immune deficiency, active immunosuppressive treatment, transplantation of major organs, or respiratory, intrathoracic, laryngeal, or breast cancer in the baseline window, and (5) maintained health plan enrollment within 1 year prior to and after the index date.

### Symptom Keyword Selection

A list of phrases or terms relevant to cough, dyspnea, wheezing, and chest tightness was compiled based on the phrases or terms published in previous literature [8-12,14] and ontologies found in the Unified Medical Language System [30] relevant to the 4 symptoms. The list was then reviewed and enriched by the study clinicians and further enhanced by the manual data annotation processing (refer to the “Data annotation process” subsection below). In addition, for each of the study terms and phrases, synonym terms, or misspelled word corrections were performed by manually examining the top 100 similar words derived from a trained deep learning word2vec model [31] based on the study corpus. The compiled phrases and terms of the 4 symptoms are summarized in Table S1 in Multimedia Appendix 1.

### Extraction and Preprocessing of Study Notes

Clinical notes and documented patient and provider telephone or email communications within 2 years before the index date (referred to as “notes” hereafter) for each study participant were extracted from the KPSC EHR system. Only the notes associated with certain medical encounters (eg, office visits), note types (eg, progress notes), and department specialties (eg, allergy) (Table S2 in Multimedia Appendix 1) were extracted. The rest (eg, physical therapy encounters) were excluded because they are unlikely to contain information relevant to the symptoms of interest. The selected notes were then preprocessed based on the following steps: (1) lowercase conversion, sentence splitting, section detection, and word tokenization [32]; (2) removal of nondigital or nonletter characters except for space, period, comma, question mark, and semicolon; (3) standardization of the abbreviated symptom phrases or terms and correction of misspelled words (Table S3 in Multimedia Appendix 1) based on the word2vec models [31], supplemented by an internal spelling correction algorithm developed in previous studies [11-13].

### Training Dataset, Validation Dataset, and Implementation Dataset

A set of 9600 notes, each containing at least one relevant phrase or term described in Table S1 in Multimedia Appendix 1, was randomly selected from the retained study notes described in the above section. These notes were randomly divided into 12 batches, each containing 800 notes (200 notes for each symptom of interest). The first 10 batches (with 8000 notes) were used for training the study algorithm (training datasets), and the last 2 batches (with 1600 notes) were used for validation of the algorithm’s performance (validation dataset). Notes not used for training or validation formed the study implementation dataset.

### Data Annotation Process

The notes of both training and validation datasets were manually reviewed by trained research annotators to indicate the presence or absence of the 4 symptoms based on the inclusion and exclusion criteria listed in Table S4 in Multimedia Appendix 1. The annotation process was based on a computer-assisted approach. First, each training and validation dataset was exported into an MS Excel spreadsheet with the highlighted prespecified phrase terms listed in Table S1 of Multimedia Appendix 1. Second, the annotators reviewed the processed notes and documented the presence or absence of each of the 4 symptoms for each sentence. Third, any undeterminable notes were adjudicated by the study clinicians and fully discussed during weekly study team meetings until a consensus was reached.

The validation dataset was double-reviewed (ie, 2 annotators independently reviewed the same set of notes). The results from the 2 annotators were compared, and inconsistencies were discussed until a consensus was reached. If the annotators did not reach a consensus, the note was reviewed and adjudicated by the study clinicians. The adjudicated results were considered the gold standard for training and validating the NLP algorithms.

The agreement, defined by the percentage of notes with identical results, and the κ coefficient [33] estimated against the double-annotated validation dataset were calculated to assess the interrater reliability among the 2 annotators.

### Rule-Based NLP Algorithm Development

We used the 10 annotated training datasets to develop the rule-based NLP algorithms via an iterative process to determine the presence or absence of the 4 symptoms of interest at the sentence level. First, the notes were searched for the phrases or terms and patterns that indicated the presence or absence of each symptom (Table S1 in Multimedia Appendix 1). In addition, any notes meeting the conditions listed in Table S4 in Multimedia Appendix 1 were identified and excluded from further processing. The algorithm was then developed to identify the patterns of the presence or absence of each symptom for each sentence. A list of negated terms (eg, denied, negative for), uncertain or probable terms (eg, likely), definite terms (eg, positive for), history terms (eg, a couple of months before), nonpatient person terms (eg, referring to a family member or friend) and general descriptions (eg, please return if you experience any following symptoms) were compiled from the training datasets. The compiled terms were refined via the repeated test-revise strategy against the manually annotated results within each training dataset until the algorithm performance reached a reasonable threshold (ie, precision >90%). The discordant cases between the algorithm and manually annotated results for each subset were further reviewed and adjudicated among the annotators and the rest of the study team until a consensus was reached.

The rules to determine the presence or absence of each symptom at the sentence level were summarized in Table S5 in Multimedia Appendix 1. Subsequently, the sentence-level results were combined to form the note-level results for each symptom of interest. The classification at the note level was determined as “Yes” if at least one sentence in the note was deemed as “Yes.” Otherwise, it was classified as “No.”

### Transformer-Based Deep Learning NLP Algorithm Development and Validation

To enhance the performance of the rule-based NLP algorithm, we used the BERT architecture [17] to develop and validate transformer-based NLP algorithms for each of the symptoms of interest. The process is described below.

We used the core learning objective masked language modeling (MLM) and followed the BERT procedure [17] for feature engineering and pretraining. A set of vocabulary words was constructed and trained from the 9600 annotated clinical notes. The clinical notes were then encoded and embedded into numerical vectors for feature pretraining. About 20% of the tokens in the notes were randomly selected for the pretraining MLM task. The parameters used for optimizing the MLM are summarized in Table S6 in Multimedia Appendix 1.

The optimized pretrain model was then used to train further and classify the 4 study symptoms separately. For each symptom, we developed and trained the BERT classification model by using the annotated training dataset via 5-fold cross-training-validation and the Adam optimizer approach [34]. The training dataset was randomly split into 5 equal subsets. Four out of 5 subsets were used as the training, and the other was used for internal validation, until every subset was used once for internal validation. The parameters used for tuning the model were summarized in Table S6 in Multimedia Appendix 1. The model used the default probability threshold of .5 to determine the classification for each sentence and each symptom (Yes when *P*≥.5; No when *P*<.5).

The final model’s discriminative power for each symptom was evaluated by the area under the receiver operating characteristic curve (AUC). The results were averaged across the internal validation and external testing datasets.

### Hybrid Algorithms

Finally, the rule- and transformer-based NLP algorithms were consolidated to generate hybrid algorithms. The results of the rule-based NLP algorithm were modified by the estimated probabilities derived from the transformer-based NLP algorithm. The cutoff threshold values for each symptom group were summarized in Table S7 in Multimedia Appendix 1. For each symptom, we determined 2 cutoff thresholds of probability generated by the transformer-based NLP algorithm to modify the results classified by the rule-based algorithm, one was used for the group classified as No by the rule-based algorithm, and the other was used for the group classified as Yes by the rule-based algorithm. These optimizing thresholds were obtained by maximizing the *F*_1_-score against the validation dataset via increasing the threshold value of the Yes group from 0 to 0.5 and decreasing the threshold value of the No group from 1 to 0.5.

### Evaluation of NLP Algorithms

The NLP algorithms were validated against manually annotated notes at both sentence and note levels. For each symptom, the numbers of true positive (TP), false positive (FP), true negative, and false negative (FN) cases were used to estimate the sensitivity (or recall), positive predictive value (PPV) (or precision), and the overall *F*_1_-score, a harmonic balance measurement of PPV and sensitivity. Sensitivity was defined as the number of TP divided by the total number of symptoms ascertained by the annotators (TP+FN). PPV was defined as the number of TP divided by the total number of symptoms identified by the computerized algorithm (TP+FP). The *F*_1_-score was calculated as: (2×PPV×sensitivity)/(PPV+sensitivity).

### Discrepancy Analysis

For each symptom, the discordant results at both sentence and note levels between the rule-based algorithm, transformer-based algorithm, and adjudicated chart review against the validation dataset were analyzed. The number of false positive and false negative cases for each comparison was summarized in detail.

### Computational Environment and Implementation of the Consolidated NLP Algorithm

The study was conducted via Python 3.10 (Python Software Foundation) programming on a dedicated machine learning Lambda workstation with 1 TB memory, an AMD Threadripper Pro 3975WX with 32 cores @ 3.50 GHz processors, and 4 RTX A6000 GPUs (graphics processing units; each with 49 GB memory). We followed the transformer-based BERT model requirements described on GitHub [35] to install all necessary packages for the model development and implementation. The BERT model feature pretraining, asthma symptom classification training, and implementation were executed simultaneously across 4 GPUs. The processing time for BERT pretraining and symptom classification training varied from 10 to 20 hours, depending on model hyperparameters and the number of GPUs used. The final NLP algorithm required approximately 140 hours to process the implementation dataset and generate results.

### Ethical Considerations

The KPSC Institutional Review Board reviewed and approved the study protocol with a waiver of the requirement for informed consent (approval number 13,414). The study complied with the Health Insurance Portability and Accountability Act, with data access restricted to authorized personnel.

## Results

### Summary of the Study Notes

A total of 11,374,552 eligible study notes and corresponding 128,211,793 sentences were retrieved during the study period. The number of sentences and words per note in the training, validation, and implementation datasets is summarized in Table 1. The 3 datasets had a similar number of words per sentence (mean values ranging from 12.6, SD 21.9, to 16.3, SD 25.2); however, the number of sentences per note in the implementation dataset (mean 11.2, SD 18.4) was smaller than those in the training dataset (mean 48.4, SD 51.8) and the validation dataset (mean 42.8, SD 37).

**Table 1.** Description of the study datasets.

Datasets	Total notes, n	Total sentences, n	Sentences per note, mean (SD)	Words per note, mean (SD)	Words per sentence, mean (SD)
Training	8000	380,363	48.4 (51.8)	612.1 (560.8)	12.6 (21.9)
Validation	1600	68,344	42.8 (37)	684.9 (601.4)	16 (27.5)
Implementation	11,364,952	127,763,086	11.2 (18.4)	183.6 (303.8)	16.3 (25.2)

### Interrater Reliability of the Two Annotators Against the Validation Dataset

The agreement and κ coefficient between the two annotators against the validation dataset at both sentence and note levels are summarized in Table S8 in Multimedia Appendix 1. The agreement ranged from 99.82% (dyspnea) to 99.97% (chest tightness) at the sentence level and 96.69% (cough) to 98.19% (chest tightness) at the note level. The κ coefficient ranged from 0.94 to 0.97 at the sentence level and 0.91 to 0.93 at the note level.

### Performance of the Transformer-Based Models

The performance of the BERT models was optimized at word sequence length=512, learning rate=1e-5, and batch size=32. Table 2 summarizes the AUC for each dataset and symptom. The performance was similar across these datasets for each symptom; all AUCs were >0.99.

**Table 2.** The mean and SD of area under the receiver operating characteristic curve of the 5-fold cross-training-validation Bidirectional Encoder Representations from Transformers models and the corresponding area under the receiver operating characteristic curve (AUC) on validation dataset for the 4 asthma-related symptoms.

Symptom	AUC		
	Training, mean (SD)	Internal validation, mean (SD)	Validation, mean (SD)
Cough	0.9989 (0.0002)	0.9975 (0.0008)	0.9986
Dyspnea	0.9963 (0.0013)	0.9935 (0.0021)	0.9973
Wheezing	0.9974 (0.0025)	0.9957 (0.0025)	0.997
Chest tightness	0.9988 (0.0007)	0.9969 (0.0026)	0.9971

### Performance of the NLP Algorithms

Table 3 summarizes the performance of the rule-based, transformer-based, and hybrid algorithms based on the 1600 notes in the validation dataset. Both rule- and transformer-based algorithms yielded a precision (PPV) and recall (sensitivity) of over 90% for all 4 symptoms at sentence and note levels.

**Table 3.** The computerized model’s performance against the adjudicated chart review results in the validation data set at the sentence level (n=68,344) and the note level (n=1600).

Symptom	PPV^a^, %	Sensitivity, %	*F*_1_-score
Sentence level			
Rule-based			
Cough	96.95	93.74	0.953
Dyspnea	96.55	93.75	0.951
Wheezing	96.52	94.69	0.956
Chest tightness	97.41	93.89	0.956
BERT^b^			
Cough	95.9	94.7	0.953
Dyspnea	91.65	90.4	0.910
Wheezing	93.06	94.12	0.935
Chest tightness	93.73	95.56	0.946
Hybrid			
Cough	97.17	95.95	0.966
Dyspnea	96.86	93.9	0.954
Wheezing	96.53	94.88	0.957
Chest tightness	97.42	94.72	0.961
Note level			
Rule-based			
Cough	96.9	97.2	0.970
Dyspnea	97.15	97.15	0.972
Wheezing	96.44	96.44	0.964
Chest tightness	97.77	95.64	0.966
BERT^b^			
Cough	96.32	97.67	0.970
Dyspnea	91.64	93.73	0.927
Wheezing	92.5	95.79	0.941
Chest tightness	93.66	96.73	0.952
Hybrid			
Cough	97.7	99.07	0.984
Dyspnea	97.71	97.15	0.974
Wheezing	96.76	96.76	0.968
Chest tightness	97.42	96	0.967

a PPV: positive predicted value.

b BERT: Bidirectional Encoder Representations from Transformers.

For the rule-based algorithm, the PPV ranged from 96.52% (wheezing) to 97.41% (chest tightness) at the sentence level and 96.44% (wheezing) to 97.77% (chest tightness) at the note level; sensitivity ranged from 93.74% (cough) to 94.69% (wheezing) at the sentence level and 96.44% (wheezing) to 97.2% (cough) at the note level. The *F*_1_-score of the 4 symptoms was >0.95 at both sentence and note levels.

For the transformer-based algorithm, the PPV ranged from 91.65% (dyspnea) to 95.% (cough) at the sentence level and 91.64% (dyspnea) to 96.32% (cough) at the note level; sensitivity ranged from 90.4% (dyspnea) to 95.56% (chest tightness) at the sentence level and 93.73% (dyspnea) to 97.67% (cough) at the note level. The *F*_1_-score ranged from 0.91 (dyspnea) to 0.953 (cough) at the sentence level and 0.927 (dyspnea) to 0.97 (cough) at the note level.

For the hybrid algorithm, the PPV ranged from 96.53% (wheezing) to 97.42% (chest tightness) at the sentence level and 96.76% (wheezing) to 97.42% (chest tightness) at the note level; sensitivity ranged from 95.95% (cough) to 93.9% (dyspnea) at the sentence level and 96% (chest tightness) to 99.07% (cough) at the note level. The corresponding *F*_1_-score of all 4 symptoms was >0.95 at both the sentence and note levels.

The consolidated hybrid algorithm resulted in superior PPV and sensitivity for all symptoms at both sentence and note levels, except that chest tightness had a slightly lower PPV (vs the rule-based algorithm) and a bit lower sensitivity (vs the transformer-based algorithm) at the note level.

### Discrepancy Analysis

The discrepancy between the rule-based algorithm, transformer-based algorithm, and the adjudicated annotated results is summarized in Table S9 in Multimedia Appendix 1. Although the majority of sentences and notes were correctly classified by both the rule- and transformer-based algorithms, a small number of notes were incorrectly classified by both algorithms (either FP or FN) for each symptom, and also a small number of notes were correctly classified by the rule-based algorithm but not the transformer-based algorithm or vice versa. Examples of each symptom misclassification by either rule-based or transformer-based algorithm were provided in Table S10 in Multimedia Appendix 1.

### Implementation of the Consolidated Algorithm

The results of the implementation dataset by the consolidated algorithm are summarized in Table 4. Of these notes, at least one symptom was identified in 1,663,450/127,763,086 (1.3%) sentences and 858,350/11,364,952 (7.55%) notes, respectively. Cough had the highest percentage at both sentence (1,363,713/127,763,086, 1.07%) and note (660,685/11,364,952, 5.81%) levels while chest tightness had the lowest one at both sentence (141,733/127,763,086, 0.11%) and note (64,251/11,364,952, 0.57%) levels. The percentage of 2, 3, and 4 symptoms was 0.38% (484,050/127,763,086), 0.19% (241,616/127,763,086), and 0.03% (36,057/127,763,086) at the sentence level and 1.85% (209,805/11,364,952), 0.71% (901,727/11,364,952), and 0.1% (10,954/11,364,952) at the note level, respectively.

**Table 4.** Presence of symptoms identified by the computerized algorithms based on the study implementation data set at both sentence and note levels.

	Sentence level (n=127,763,086), n (%)	Note level (n=11,364,952), n (%)
Symptom		
Cough	1,363,713 (1.07)	660,685 (5.81)
Dyspnea	678,778 (0.53)	312,703 (2.75)
Wheezing	554,679 (0.43)	224,918 (1.98)
Chest tightness	141,733 (0.11)	64,251 (0.57)
Any of above symptoms	1,663,450 (1.3)	858,350 (7.55)
Number of symptoms^a^		
1	901,727 (0.71)	556,821 (5)
2	484,050 (0.38)	209,805 (1.85)
3	241,616 (0.19)	80,770 (0.71)
4	36,057 (0.03)	10,954 (0.1)

a The number of mutual symptoms present.

## Discussion

In this study, we successfully developed a hybrid NLP framework combining the results of rule- and transformer-based NLP algorithms to capture 4 asthma-related symptoms from clinical notes and patient and provider communications. The validated models demonstrated high accuracy, with precision (PPV) and recall (sensitivity) exceeding 90% at both the sentence and note levels.

Both the rule- and transformer-based algorithms performed well, with some notable differences. The transformer-based algorithm generally yielded higher recall (sensitivity) at both sentence and note levels, except for dyspnea and wheezing, while the rule-based algorithm exhibited superior precision (PPV). Previous research has similarly shown that rule-based models can outperform machine learning or deep learning approaches in domain-specific tasks [36,37]. For example, a systematic meta-analysis study of NLP models for classifying EHR documentation in mental health care found that rule-based models achieved higher precision (average: 88.1% vs 79.1%), recall (average: 83.3% vs 73.3%) and *F*_1_-score (average: 0.845 vs 0.718) compared to machine learning models [36]. Likewise, another study demonstrated that rule-based models were more effective than transformer-based models in performing domain-specific communication tasks [37]. These findings suggest that approach selection should be guided by the specific needs of a study, available resources, and performance metrics. For clinical applications where minimizing false positives is crucial, such as decision support systems and clinical trials, rule-based NLP may be preferable [38].

Hybrid approaches that combine rule-based and machine learning algorithms can leverage the strengths of both algorithms to create a more robust, flexible, and accurate solution. It has been shown to yield higher performance in various applications, such as identifying asthma control factor [25], identifying suicide ideation and suicidal attempts [39], extracting negative schizophrenia symptoms [40], mining occupational data [41], and deidentifying radiology reports [42]. In this study, we demonstrated that the hybrid approach leveraging both approaches can further enhance performance, optimizing both precision and recall in asthma symptom extraction. Given the increasing availability of computational resources, hybrid models may provide a balanced and effective solution for NLP tasks in health care settings.

Despite the growing adoption of transformer-based models in clinical NLP, their lack of interpretability remains a significant challenge. The complex “black box” architecture of deep learning models makes it difficult to understand how specific predictions are generated [43,44]. For example, the trained transformer-based model generated a 0.825 predicted value of dyspnea for the sentence “*overnight events/subjective: patient feeling much better since admission, forgot to put on her oxygen this morning and not complaining of shortness of breath*” and a 0.033 predicted value of chest tightness for the sentence “*no wheezing or dyspnea but chest feels tight.*” In addition, transformer-based models require a large amount of labeled data for training and substantial computational resources for implementation. In contrast, rule-based approaches offer greater transparency, allowing researchers to analyze misclassification cases and refine decision rules more effectively. Future research should explore post hoc explainability techniques, such as feature importance analysis [45], integrated gradients [46], surrogate models [47], and Shapley Additive Explanations [48], to improve the interpretability of deep learning models in clinical applications.

Extracting symptoms from free-text clinical notes presents multiple challenges. First, symptoms may be documented in various sections of a note, including past medical history, review of systems, problem lists, instruction, sign and symptom warning, questionnaire, symptom checklist, allergy and side effects, current or past medication, procedure, diagnosis, or chief complaint. Each research study needs to determine which sections are appropriate for extraction. For example, if problem lists are outdated, including symptoms from this section may introduce error. In addition, negation detection remains a critical factor. In some cases, negations apply to a single symptom (eg, “no wheezing, mild SOB”), while in others, they apply to multiple symptoms (eg, “denied fever, chills, wheezes, GERD, or any new medication”). Accurately handling such cases is essential for improving NLP performance.

In this study, 16.7% (1600/9600) of annotated notes were double-reviewed by 2 independent annotators, yielding higher agreement (>95%) and stronger κ coefficients (>0.91) than those in previous studies [13]. Double annotation minimizes inconsistencies and ensures a robust gold standard for training and validation. In addition, we recommend that future NLP studies include a training period for annotators, during which study investigators with medical expertise review a subset of notes together with the annotators. This process helps establish consistent annotation criteria before formal chart review begins. In addition, creating a detailed annotation guide can improve accuracy and reproducibility.

Our study has several limitations. First, the accuracy of symptom extraction depends on how symptoms were documented in the EHR. Incomplete or inaccurate documentation of symptoms in the EHR may lead to misclassification. Second, we excluded symptoms documented in notes that also mentioned anxiety, as symptoms could be attributed to anxiety rather than asthma. This approach may have led to the omission of some true asthma-related symptoms. Third, the rule-based algorithm relied on a predefined lexicon, which may not fully capture all variations in symptom descriptions. Expanding the lexicon with additional samples from diverse datasets could improve performance. Similarly, the rule-based approach used a fixed word distance threshold for certain symptoms (eg, allowing a maximum of 3 words between “tightness” and “chest”), which may have resulted in missed cases when symptoms were described in less conventional ways. Fourth, it is challenging for the transformer-based algorithm to rule out the positive symptom description due to the study-specific exclusion criteria and rules. More extensive sample training could improve the predictions [49]. In addition, the current feature pretraining BERT model was trained based on the annotated dataset rather than the entire study notes due to limited GPU memory. Finally, our training dataset consisted only of notes containing predefined symptom keywords, this selection process may have introduced bias by excluding alternative descriptive patterns.

Although this study focused on symptom extraction in adults with mild asthma, asthma symptom descriptions are unlikely to differ significantly across severity levels or between pediatric and adult populations. In addition, our NLP models were developed using clinical notes from a single integrated health care system. When applied to other health care settings, modifications may be required to account for differences in note structure and terminology.

In conclusion, the study successfully developed and validated a hybrid NLP algorithm to extract asthma-related symptoms from unstructured clinical notes with high accuracy. The algorithm can be used to facilitate early asthma detection and predict exacerbation risk. Future research should explore external validation across different health care systems, improve model interpretability, and refine hybrid NLP approaches to optimize both precision and recall in clinical text mining applications.

## Supplementary material

10.2196/69132 Multimedia Appendix 1 The detailed supplementary materials of the hybird natural language processing algorithm.

## Abbreviations

AUC: area under the receiver operating characteristic curve
BERT: Bidirectional Encoder Representations from Transformers
EHR: electronic health record
FN: false negative
FP: false positive
GPU: graphics processing unit
*ICD*: *International Classification of Diseases*
KPSC: Kaiser Permanente Southern California
MLM: masked language modeling
NLP: natural language processing
PPV: positive predictive value
TP: true positive
