# Supplementary material for: Identifying Asthma-Related Symptoms From Electronic Health Records Using a Hybrid Natural Language Processing Approach Within a Large Integrated Health Care System: Retrospective Study
Source: JMIR AI. 2025 May 2;4:e69132. doi: 10.2196/69132 (PMC12231518; doi:10.2196/69132)
Supplement: Multimedia Appendix 1 [file ai-v4-e69132-s001.docx]

Table S1. Phrases or Terms used for identifying asthma related symptoms.

| **Symptom** | **Phrases/terms^¶^** |
| --- | --- |
| Cough | cough, coughed, coughing, tussis, tussive |
| Dyspnea | dyspnea, shortness breath, shortness of breath, short of breath, sob, labor breathing, labor respiration, difficulty breathing, breathing difficulty, trouble breathing, gasping, hyperpnea, breathlessness, respiratory distress, cannot breath, can’t breath, be out of breath, hard to breath, harder to breath, quick breathing, lack of breath, rapid breathing, frequent shallow breathing, hypoxemic, bronchogram |
| Wheezing | wheezing, wheeze, whooshing, whizzing, rasp breathing, noisy breathing, whistle breathing, asthmatic breathing, stertor |
| Chest tightness | chest tightness, tight chest, chest constraint, chest symptom tightness, oppression chest, chest oppression, tight breathing, chest squeezing, squeezing chest, tightness related to chest area |

¶ Also search lemma variants (adjective, adverb, plural form and verb tenses) and abbreviations of these words, where applicable.

Table S2. Inclusion of encounter, specialty and clinical note types used for identifying asthma related symptoms.

| **Encounter** | Allied Health/Nurse Visit, Call Center New Member, Call Center Nurse Advice, Call Center Telephone Encounter, Clinical Documentation, E-Visit, External Hospital Admission, Home Visit, Hospital Encounter, KP On Call Telephone, Message,  Non Nursing - Allied Health, Nursing Facility, OB Office Visit, OB Video Visit, Office Visit, Patient Message, Patient Outreach, Patient Self-Triage, Telephone, Telephone Appointment Visit, Telephone Appointment Visit - Allied Health/Nurse Visit, Transitional Care Mgmnt-Office Visit, Transitional Care Mgmnt-Telephone, Video Visit, Video Visit Allied Health |
| --- | --- |
| **Note type** | Addendum Note, Consults, ED Notes, ED Provider Notes, ED Provider Triage Note, H&P, H&P (View-Only), Initial Assessments, Interval H&P Note, Multi-Discipline Progress Note, Nursing Note, Progress Notes, Rapid Response, Telephone Encounter, UC Nurse Notes, Null/missing |
| **Specialty** | Adolescent Medicine, Allergy, Asthma and Immunology, Emergency Medicine, Family Practice, Home Health Care, Hospital Medicine, Internal Medicine, Nursing, Advice, Obstetrics, Obstetrics, Gynecology, Occupational Medicine, Otolaryngology, Pediatric Pulmonology, Pediatric Urgent Care, Pediatrics, Population Care Management, Pulmonary Diseases, Respiratory Therapy, Urgent Care, Null/missing |

Table S3. Corrections of mistyped or concatenating asthma-related symptom keywords/terms.

| Mistyping or concatenating words/terms | Corrected words/terms |
| --- | --- |
| breathre, breah, rbreath, bretah, breaeth, berath, breeath, breatth, breaht, bteath, beath, bretath, reath, nreath, breth, breathh, brearh, brearh, brathe, bretha, brewath, breawth, breadth, breat, breathq, brrath | breath |
| breathand | breath and |
| breathdecreased | breath decreased |
| breathfevers | breath fevers |
| breathholding | breath holding |
| breathstates | breath states |
| breathsince | breath since |
| breathwith | breath with |
| brething, breating, breathin, breasthing, brreathing, rbeathing, breathign,berthing,breathng, breadthing, breathi, breahing, braething, breathingin, breahting, breading, breathining | breathing |
| breathingmostly | breathing mostly |
| breathingno | breathing no |
| breathingpatient | breathing patient |
| breathingpersistent | breathing persistent |
| breathingor | breathing or |
| caughing, couphing, coughin,coghing, | coughing |
| complaintshortness | complaint shortness |
| chets, chesst, chset, chst, | chest |
| denieschest | denies chest |
| forchest | for chest |
| nochest | no chest |
| withchest | with chest |
| onsetchest | onset chest |
| couph, couhg, jcough, courgh, coughx, coufh, coug, crough, cugh, | cough |
| deniesshortness | denies shortness |
| dif breathing | difficulty breathing |
| difficutly, dificulty, diffiulty, diffficulty, dififculty, diffuclty, diffuculty,diffiuclty, difficultiea, difficulities, difficlty, diffciulty, difficulat, difficully, difficulat, diificulty, difficuty, tdifficulty, difficu,difficul, odifficulty, diffivculty, diffuclty, diffuculty, ifficulty. diffi | difficulty |
| hasdifficulty | has difficulty |
| developdifficulty | develop difficulty |
| indifficulty | in difficulty |
| nodifficulty | no difficulty |
| worrydifficulty | worry difficulty |
| dyspea, dyspena, dyapnea, dypsnea, dypnea, dyspne, yspnea, dyspna, dyuapnea | dyspnea |
| forcough | for cough |
| laboured | labored |
| mildshortness | mild shortness |
| ofbreath | of breath |
| orshortness | or shortness |
| respitory, respiatory, raspatory, respritory, repistory, repistory, respiritory, respiratiory, respiraty, respirtory | respiratory |
| dsitress, distres, distess, disress, disdtress, distresss, ditress, istress, distressy | distress |
| shorness, shorntess, shotnes, sortness, shortess, shortneess, shorthness, shotness, shprtness, shrotness, shortnees, shrtness, shortnfess, shorteness, shortnss, shortnbess, shrontess, shrortness, shortneness, shourtness, shortnness, shortrness, hortness, shorntes, shortnress, shortnesws, shortnerss, shoretness, shotrness, shortnedss, sjhortness, shothess, shoertness | shortness |
| shortness if breath | shortness of breath |
| sobx, soboe | sob |
| symptomsshortness | symptoms shortness |
| history or shortness of breath | history of shortness of breath |
| thickcough | thick cough |
| tighness, thightness, tighntess, tightnes, tighteness, tenseness, tightn, tightenss, thighness, tghnees, tightning, tigthness, thightness, tighntess, tightnes, tightnesss, ithgtness, tightenss | tightness |
| weezing, wizzing, whezing, wheezinf, whizzing, whezzing, | wheezing |
| whezzingor | wheezing or |
| wheezingpost | wheezing post |
| withdyspnea | with dyspnea |
| yesshortness | yes shortness |

Table S4. Exclusion criteria for asthma-related symptoms

| **Criteria** | **Condition** |
| --- | --- |
| All symptoms | - Symptom appeared in patient active problem list - Symptom appeared in past medical history - Symptom appeared in instruction (e.g., patient instruction, medication instruction, discharge instruction) - Symptom indicated by medication usage but prescribed as prn or as needed or initiated by provider - Symptom indicated by side effects, allergy section - Symptom associated with anxiety - Symptom in subject line of patient message/communication - Symptom occurred before one month prior to the note date |
| Chest tightness | - Tightness not in the location other than chest area |

Table S5. Rules to determine the presence of symptom for each note at the sentence level.

| A symptom was classified as “No” for any of the situations | - Sentence met one of exclusion criterion in Table S4 of the Multimedia Appendix. - The sentence did not contain any defined terms listed in Table S1 of the Multimedia Appendix. - The negated description was associated with defined terms listed in Table S1 of the Multimedia Appendix. Examples included “patient denied chest tightness”, “no cough”, and “negative for wheezing”. - A probable or uncertain description was associated with the study symptom. For example, “patient with difficulty breath and likely wheezing”. - The description of the symptom did not refer to an actual situation. For example, “report to ER right away if you experience severe short of breath ”, and “severe allergy reactions such as shortness of breath”. - The symptom was associated with a historical term or date relative to the clinical note date. For example, “had dry cough in the past year”, and “had chest tightness and wheezing in last year”. - The symptom description was related to family history, such as “family history: daughter with cough and occasional wheeze”. - Someone other than the patient had a symptom. For example, “my friend tell me she had wheezing”. - The sentence only consisted of a symptom term. So, a decision could not be reached whether this instance was positive for the symptom without additional other description associated with it. |
| --- | --- |
| A symptom was classified as “Yes” for any of the situations | - Marked as “Yes”, “x” or “+”. A symptom was classified as “yes” if the response to a symptom question was affirmative, or if the symptom was marked on the symptom list. - The symptom was listed under the diagnosis section, chief complaint section, reason for visit section, symptom section and history of present illness section of the clinical note. For example, “reason for visit: fever, cough, wheezing”, “chief complaint: shortness of breath and wheezing”. - The symptom was described as treated or indicated by medication initiated by patient. For example, “patient request nebulizer or inhaler for cough with wheezing”. - The symptom was documented or reported to be present at the time of visit or messaging. For example, “persistent productive cough for over one week”, “had been having fevers, cough, and wheezing since last month”. - The sentence contained a definite term associated with a study interested symptom. Examples included “positive for cough and wheezing ”, “patient complaint of sob”, and “patient present to the emergency department with wheezing and nonproductive cough”. |

Table S6. Hyperparameters used for BERT model development.

| Task | Vocabulary word size | Word token masked rate | Sequence length | Learning rate | Min-batch size | Training epochs |
| --- | --- | --- | --- | --- | --- | --- |
| Masked Language model | 50400 | 20% | 128, 256, 512 | Model auto-tunning | 8, 16, 32, 64 | 10, 20, 40 |
| Symptom classification | 50400 | NA | 128, 256, 512 | 1e-4, 1e-5, 2e-5, 5e-5 | 8, 16, 32, 64 | 1, 2, 5 |

BERT: bidirectional encoder representations from transformers

Table S7. The cutoff threshold of probability used to combine the rule-base NLP algorithm and BERT algorithm for the four asthma-related symptoms.

| Symptom | Rule-based classification | Deep learning-based probability threshold | Final classification |
| --- | --- | --- | --- |
| Cough | Yes | P ≥ 0.14 | Yes |
|  |  | P < 0.14 | No |
|  | No | P ≥ 0.82 | Yes |
|  |  | P < 0.82 | No |
| Dyspnea | Yes | P ≥ 0.03 | Yes |
|  |  | P < 0.03 | No |
|  | No | P ≥ 0.98 | Yes |
|  |  | P < 0.98 | No |
| Wheezing | Yes | P ≥ 0.02 | Yes |
|  |  | P < 0.02 | No |
|  | No | P ≥ 0.99 | Yes |
|  |  | P < 0.99 | No |
| Chest tightness | Yes | P ≥ 0.35 | Yes |
|  |  | P < 0.35 | No |
|  | No | P ≥ 0.91 | Yes |
|  |  | P < 0.91 | No |

Table S8. Agreement and kappa coefficient between the two annotators at both sentence and note levels against validation dataset.

| **Symptom** | **Agreement (%)** | **Kappa coefficient and 95% confidential interval** |
| --- | --- | --- |
| *Sentence level* |  |  |
| Cough | 99.95 | 0.97 [0.95, 0.98] |
| Dyspnea | 99.82 | 0.95 [0.94, 0.96] |
| Wheezing | 99.88 | 0.94 [0.92, 0.95] |
| Chest tightness | 99.97 | 0.97 [0.96, 0.98] |
| *Note level* |  |  |
| Cough | 96.69 | 0.93 [0.91, 0.94] |
| Dyspnea | 97.12 | 0.91 [0.89, 0.94] |
| Wheezing | 98.06 | 0.93 [0.91, 0.96] |
| Chest tightness | 98.19 | 0.93 [0.91, 0.96] |

Table S9. Comparison of symptom classifications by ruble-based approach versus BERT approach against the adjudicated chart review results of the validation dataset at both sentence (n=68344) and note (n=1600) levels.

|  | Cough | Dyspnea | Wheezing | Chest tightness |
| --- | --- | --- | --- | --- |
| *Sentence level* |  |  |  |  |
| Rule-based: TP, BERT: TP | 1230 | 570 | 480 | 329 |
| Rule-based: TN, BERT: TN | 66904 | 67623 | 67775 | 67959 |
| Rule-based: FP, BERT: FP | 13 | 11 | 13 | 7 |
| Rule-based: FN, BERT: FN | 29 | 18 | 12 | 7 |
| Rule-based: TP, BERT: FN | 43 | 45 | 19 | 9 |
| Rule-based: FN, BERT: TP | 56 | 23 | 16 | 15 |
| Rule-based: FP, BERT: TN | 27 | 11 | 5 | 2 |
| Rule-based: TN, BERT: FP | 42 | 43 | 24 | 16 |
| *Note level* |  |  |  |  |
| Rule-based: TP, BERT: TP | 614 | 324 | 290 | 257 |
| Rule-based: TN, BERT: TN | 921 | 1212 | 1263 | 1307 |
| Rule-based: FP, BERT: FP | 9 | 3 | 7 | 6 |
| Rule-based: FN, BERT: FN | 3 | 5 | 5 | 3 |
| Rule-based: TP, BERT: FN | 12 | 27 | 8 | 6 |
| Rule-based: FN, BERT: TP | 15 | 5 | 6 | 9 |
| Rule-based: FP, BERT: TN | 11 | 7 | 4 | 0 |
| Rule-based: TN, BERT: FP | 15 | 27 | 17 | 12 |

TP: True positive; TN: True negative; FP: False positive; FN: False negative

Table S10. Examples of misclassification by either rule-based or BERT algorithm against the adjudicated chart review results.

| Examples | Symptom | Rule-based | BERT | Chart review |
| --- | --- | --- | --- | --- |
| no sob no wheezing coughing up phlegm no fevers little ear pressure | Cough | no | no | yes |
| respiratory: denies wheezing, shortness of breath, +dry cough | Cough | yes | no | yes |
| for possible post nasal drip which can give a cough, please rinse your sinuses and use flonase otc nasal spray for few weeks | Cough | yes | no | no |
| overnight events/subjective: patient feeling much better since admission, forgot to put on her oxygen this morning and not complaining of shortness of breath | Dyspnea | no | yes | no |
| no vomiting or nausea mild shortness of breath on exertion | Dyspnea | yes | no | yes |
| 3) shortness of breath | Dyspnea | yes | yes | no |
| is there something over the counter to use, or can you flush them out? still having trouble with wheezing, not all the time | Wheezing | yes | No | yes |
| the wheezing has nearly resolved, he does not have any of his inhalers | Wheezing | no | yes | yes |
| rx meds took care of wheezing, but my throat remains bothered | Wheezing | yes | yes | no |
| no wheezing or dyspnea but chest feels tight | Chest tightness | yes | no | yes |
| associated symptoms: no fever chest tightness associated symptoms: no fever | Chest tightness | no | no | yes |
| but usually, if the cough is from asthma and heart failure, you will also get chest tightness | Chest tightness | no | yes | no |
